# Genome-wide identification and characterization of *AP2/ERF* gene superfamily during flower development in *Actinidia eriantha*

**DOI:** 10.1186/s12864-022-08871-4

**Published:** 2022-09-13

**Authors:** Quan Jiang, Zhi Wang, Guangming Hu, Xiaohong Yao

**Affiliations:** 1 CAS Key Laboratory of Plant Germplasm Enhancement and Specialty Agriculture, Wuhan Botanical Garden, the Chinese Academy of Sciences, Wuhan, 430074 Hubei China; 2 College of Life Sciences, University of Chinese Academy of Sciences, Beijing, China; 3 Institute of Fruit and Tea, Hubei Academy of Agricultural Sciences, Wuhan, 430064 China

**Keywords:** Kiwifruit, *AP2/ERF*, Phylogenetic analysis, Comparative genomics, Expression analysis, Flower development

## Abstract

**Background:**

As one of the largest transcription factor families in plants, *AP2/ERF* gene superfamily plays important roles in plant growth, development, fruit ripening and biotic and abiotic stress responses. Despite the great progress has been made in kiwifruit genomic studies, little research has been conducted on the *AP2/ERF* genes of kiwifruit. The increasing kiwifruit genome resources allowed us to reveal the tissue expression profiles of *AP2/ERF* genes in kiwifruit on a genome-wide basis.

**Results:**

In present study, a total of 158 *AP2/ERF* genes in *A. eriantha* were identified. All genes can be mapped on the 29 chromosomes. Phylogenetic analysis divided them into four main subfamilies based on the complete protein sequences. Additionally, our results revealed that the same subfamilies contained similar gene structures and conserved motifs. Ka/Ks calculation indicated that *AP2/ERF* gene family was undergoing a strong purifying selection and the evolutionary rates were slow. RNA-seq showed that the *AP2/ERF* genes were expressed differently in different flower development stages and 56 genes were considered as DEGs among three contrasts. Moreover, qRT-PCR suggested partial genes showed significant expressions as well, suggesting they could be key regulators in flower development in *A. eriantha*. In addition, two genes (*AeAP2/ERF061, AeAP2/ERF067*) had abundant transcription level based on transcriptomes, implying that they may play a crucial role in plant flower development regulation and flower tissue forming.

**Conclusions:**

We identified *AP2/ERF* genes and demonstrated their gene structures, conserved motifs, and phylogeny relationships of *AP2/ERF* genes in two related species of kiwifruit, *A. eriantha* and *A. chinensis*, and their potential roles in flower development in *A. eriantha*. Such information would lay the foundation for further functional identification of *AP2/ERF* genes involved in kiwifruit flower development.

**Supplementary Information:**

The online version contains supplementary material available at 10.1186/s12864-022-08871-4.

## Background

Transcription factors (TFs) are a class of proteins that take part in various biological processes including fruit ripening, plant growth and development, as well as biotic and abiotic stress responses such as drought, low temperature, pathogen infecting [1]. With the increasing reports of the plant genomes, numerous transcription factor families including MYB [2], bHLH [3], WRKY [1] and AP2/ERF in different species were clearly detected. Among them, AP2/ERF (APETALA 2/Ethylene Responsive Element Binding Factor) superfamily was early confirmed in *Arabidopsis thaliana*, and involved in flower and seed development [4–6]. Subsequently, all 147 genes were finally identified in *A. thaliana* [7–9]. Then, *AP2/ERF* family was gradually confirmed in diverse species and showed various numbers with different genome sizes. For example, 114 *AP2/ERF* genes were found in *Ricinus communis* (~ 336 Mb) [10] and 131 *AP2/ERF* genes were identified in peach (*Prunus persica*) (~ 228 Mb) [11].

According to the previous studies, a striking feature of all members from *AP2*/*ERF* gene family is containing at least one AP2 domain consists of about 60–70 amino acids [6]. Based on the number of AP2 domain, they are mainly regarded as *AP2* genes with variable amount of AP2 domains and *ERF* genes with only single AP2 domain [12]. *AP2*/*ERF* gene family can be divided into five subfamilies, APETELA2 (AP2), ABI3/VP1 (RAV) containing both of AP2 and B3 domain, DREB (dehydration-responsive element-binding protein), ERF (ethylene-responsive factor) subfamily and other proteins (Soloist). Additionally, DREB subfamily can be grouped into A1 to A6, and ERF subfamily can be grouped into B1- B6 as well [8]. *AP2/ERF* genes were closely related to flowering in previous studies, especially genes in the AP2 family [13, 14]. Flowering was widely recognized as a key symbol in transition from vegetative growth to reproductive growth in plants [15]. Illustrating the mechanisms of flower development and flower transition has great significance for plant adaptation to poor environment and plant breeding [16]. There is plenty of evidence of implication of *AP2/ERF* genes in flower development processes. For instance, in ‘ABC’ model of flower development, *APETALA2* (*AP2*) as an A-class gene took part in flower formation [17]. Recently, researchers have found that the expressions of two *DoAP2* genes (*DoAP2–8* and *DoAP2–10*) were down-regulated and another two *AP2* genes (*DoAP2–2* and *DoAP2–3*) were up-related during flower development, which suggested that *DoAP2* genes contribute to plant regeneration and flower development in *Dendrobium officinale* [18]. Overexpression of miR172, whose target is *AP2*-like gene, resulted in the double flower phenotype in roses [19]. Additionally, the *AP2*-like transcriptional factor TOE1 could also regulate *FT* expression to control flowering [20]. In flowering plants, *SOC1* might combine with the promoter of *CBF*s to influence flowering in cold environment [21].

Kiwifruit is a dicotyledonous, perennial, deciduous plant belonging to the genus *Actinidia* (Actinidiaceae) [22]. It has great morphological variations for stems, leaves, flowers and fruits. As a vital fruit contains huge commercial value and considerable nutritive value, it is called “the King of fruits” [23]. Kiwifruit originated from China and gradually spread to other countries [24]. In the early twentieth century, kiwifruit was beginning to be domesticated and cultivated, and some excellent cultivars were selected such as ‘Hayward’, ‘Bruno’ for *Actinidia chinensis* Planch. var. *deliciosa*, ‘Hongyang’ for *Actinidia chinensis* Planch. and ‘White’ for *Actinidia eriantha* Benth. Recently, kiwifruit breeding and genetic studies have been carried out for different purposes all over the world [25], however, there are few studies on the flower development and timing of kiwifruit. As we know, breeding early flowering cultivars to decrease the risk of frost damage is an important breeding goal. Therefore, it is very necessary to reveal the genes involved in flower development and timing. *AP2/ERFs* play a vital role in plant flower development. However, little is known about the importance of *AP2/ERF* genes in kiwifruit flowering.

**Fig. 1:**
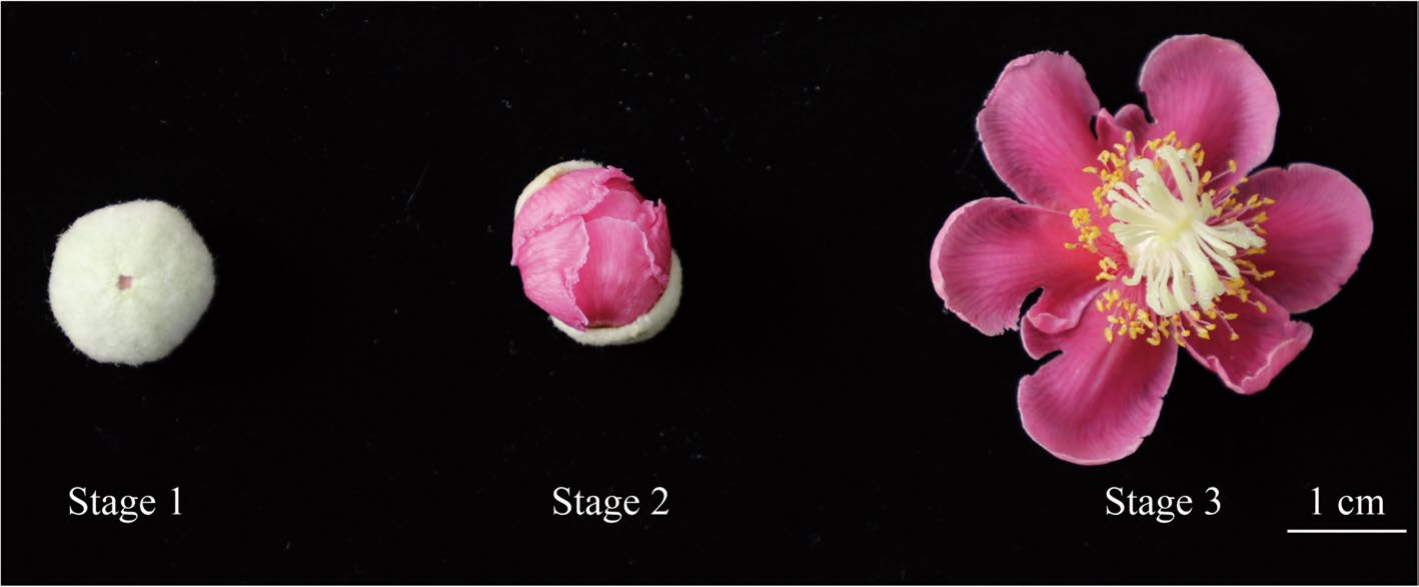
Flower tissues of *A. eriantha* sampled for RNA-seq. Stage 1: flower buds; stage 2: unopened flowers; stage 3: full-opening flowers

Recently, high-quality genomes for *A. eriantha* and *A. chinensis* were available, which provides an opportunity to reveal the tissues specificity and expression profiles of *AP2/ERF* genes in kiwifruit on a genome-wide basis [26, 27]. In this study, we systematically conducted genome-wide analysis of *AP2/ERF* gene superfamily in *A. eriantha* through investigating the sequence conservation, gene locations, gene structures and evolutionary relationships*.* In addition, we investigated the expression profiles of *AP2/ERF* genes in three flower development stages (flower buds, unopened flowers and full-opening stage) of *A. eriantha* (Fig. 1). Our study provided a unique and fundamental insight in the molecular evolution of *AP2/ERF* genes in kiwifruit and added valuable information for screening important *AP2/ERF* genes in regulating floral development and blooming in woody perennials.

## Results

### Identifications and characterizations of *AP2/ERF* family members in kiwifruit

We obtained 175 candidate genes in *A. eriantha* through the HMMER software [28]. Similarly, 191 unique protein sequences were selected in kiwifruit by BLASTP [29] program. Then, the common genes confirmed by above two methods were filtered via three common databases. Finally, 158 genes in *A. eriantha* were retained. According to their orders on the chromosomes, we renamed all the genes *AeAP2/ERF001* to *AeAP2/ERF158*. The whole proteins and coding sequences were listed in Additional file 1 Table S1.

Among them, 141 genes had one complete AP2 domain in *A. eriantha* while 17 genes had two or more domains such as *AeAP2/ERF039* which contained three domains (Additional file 2 Table S2). The length of protein in *A. eriantha* ranged from 95 aa (AeAP2/ERF040) to 783 aa (AeAP2/ERF026) with an average of 297 amino acids. Consistent with the number of amino acids, the longest coding sequence contained 2,349 bp (*AeAP2/ERF026*) and the shortest was only 288 bp (*AeAP2/ERF040*). The molecular weight (MW) ranged from 10.57 (AeAP2/ERF040) to 89.36 kDa (AeAP2/ERF026) and the isoelectric point (PI) ranged from 4.5 (AeAP2/ERF152) to 11.97 (AeAP2/ERF060).

In order to explore the evolution of *AP2/ERF* gene family in *Actinidia*, 189 *AP2/ERF* genes in *A. chinensis* renamed *AcAP2/ERF001* to *AcAP2/ERF189* were also identified and analyzed (Additional file 1 Table S1). The protein length varied from 81 (AcAP2/ERF129) to 922 aa (AcAP2/ERF052). The coding sequence length ranged from 246 (*AcAP2/ERF129*) to 2,769 bp (*AcAP2/ERF052*). MW ranged from 9.24 to 102 kDa. PI ranged from 4.51 (AcAP2/ERF080) to 10.71 (AcAP2/ERF066). The detailed information about *AP2/ERF* gene family in both *A. eriantha* and *A. chinensis* were presented in Additional file 3 Table S3.

### Classification and phylogenetic analysis of *AP2/ERF* gene family

**Fig. 2:**
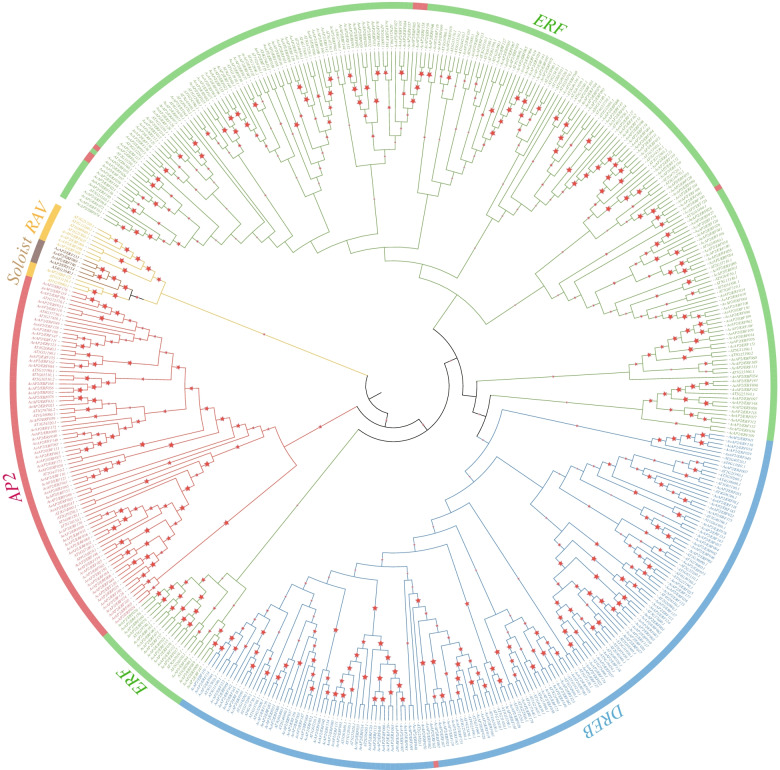
The phylogenetic tree of 347 *AP2/ERF* genes from two kiwifruit species. Different color represents different subfamily. Red pentacle means the bootstrap value

To evaluate the evolutionary relationships of *AP2/ERF* superfamily among *A. eriantha*, *A. chinensis* and *A. thaliana*, the full-length protein sequences were first aligned. Then, 494 protein sequences were used to conduct a phylogenetic tree using Neighbor-joining (NJ) method with 1,000 bootstrap replications, poisson model and pairwise deletion. Referred to classification of *AP2/ERF* genes in *A. thaliana* [9], all genes from kiwifruit were divided into four primary subfamilies referring to RAV, AP2, ERF and DREB subfamily (Fig. 2). In *A. eriantha*, 72 genes belonged to ERF subfamily and 47 genes were clustered to DREB subfamily. About 22% (34 genes) of genes were assigned to AP2 subfamily. Three genes contained one AP2 domain and a B3 domain were divided into RAV family. ERF subfamily contained most genes, followed by DREB and AP2 subfamily, and RAV subfamily was made up of the fewest genes. ERF subfamily genes accounted for 51% (96 genes) in *A. chinensis* and the rate of DREB subfamily genes was only 28% (53 genes). Then 35 genes were segmented into AP2 subfamily (Additional file 3 Table S3). In addition, four genes (*AeAP2/ERF089*, *AeAP2/ERF154*, *AcAP2/ERF133*, *AcAP2/ERF166*) were identified as Soloist in *A. eriantha* and *A. chinensis* as they had high homology with *AT4G13040.1* in *A. thaliana*. Interestingly, there were several genes belonging to the AP2 family clustered in the ERF subgroup in both species (Fig. 2).

### Structural and conserved motif analysis of the *AP2/ERF* superfamily

**Fig. 3:**
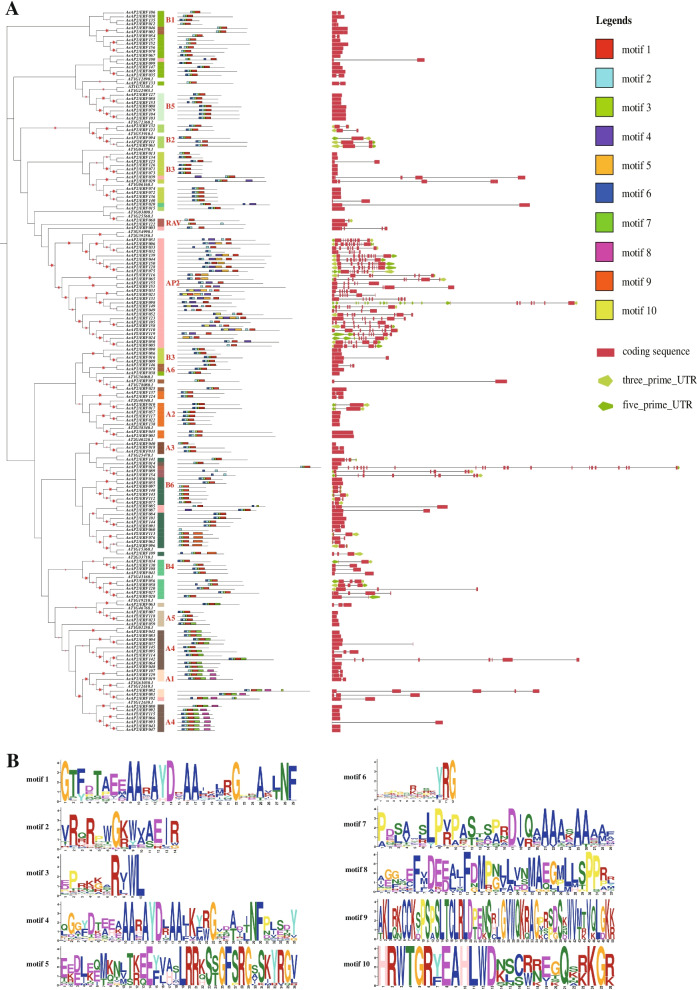
Gene structures and distributions of conserve motifs of *AP2/ERF* genes in different subfamilies in *A. eriantha*. **A** The phylogenetic tree of *AP2/ERF* genes in *A. eriantha* (left); Motifs contained in AP2/ERF proteins (middle) and the exon-intron structures of kiwifruit *AP2/ERF* genes (right). **B** Protein sequences of conserved motifs identified in *A. eriantha*. Red pentacle means the bootstrap value (20–100%)

To better understand the evolutionary relationships and structural components of *AP2/ERF* superfamily in kiwifruit, the exon–intron gene structures based on genome sequences and conserved motifs based on protein sequences were analyzed. Structural analysis suggested that not all *AP2/ERF* genes in *A. eriantha* had at least one intron, in contrast, 65 genes (41.1%) had no intron. Most genes existed one or two exons, comprised 58.8% of the total. But there are still a small subset of genes contained 9, 10 or 22 exons (Fig. 3A; Additional file 4 Table S4). Overall, the number of exons in *A. eriantha* varied from 1 to 22, while from 1 to 17 in *A. chinensis*. The genes containing the maximum number of exons were *AeAP2/ERF026* and *AcAP2/ERF056* in *A. eriantha and A. chinensis,* respectively.

Meanwhile, ten motifs were searched in 158 AP2/ERF proteins sequences in *A. erianth* (Fig. 3B), and only three motifs (motif 1, motif 2 and motif 4) were identified as locating in the AP2 domain (Additional file 5 Table S5). All AP2/ERF proteins contained motif 2 or motif 1 (except AeAP2/ERF032). However, only 22 proteins contained motif 4, and 21 out of which belonged to AP2 family (Fig. 3). The frequency of motif 3 was higher than that of motif 5, although both of them were common in 158 protein sequences. In the gene family, some motifs were only found in a subfamily. For example, motif 9 just existed in several ERF-B6 subfamily genes and motif 10 was only identified in part of AP2 subfamily (Fig. 3A).

### Cis-acting regulatory elements analysis of the *AP2/ERF* superfamily

A total of 3,992 cis-elements including 21 primary types were identified and divided into following three main parts, plant development, abiotic and biotic stress responses contained drought-inducibility, light-responsiveness, low-temperature responsiveness, defense and stress responsiveness such as wounds and salts, and plant hormone responses including SA-responsiveness, ABA-responsiveness, MeJA-responsiveness, GA-responsiveness and Auxin-responsiveness (Additional file 6 Fig. S1). Among these, the light-responsiveness was detected in all genes. Additionally, ABA-responsiveness, MeJA-responsiveness also appeared in most genes. As the important components, biotic and abiotic stresses occurred in all families. The more detailed information was listed in Additional file 7 Table S6.

### Gene distribution and gene duplication events in *A. eriantha*

**Fig. 4:**
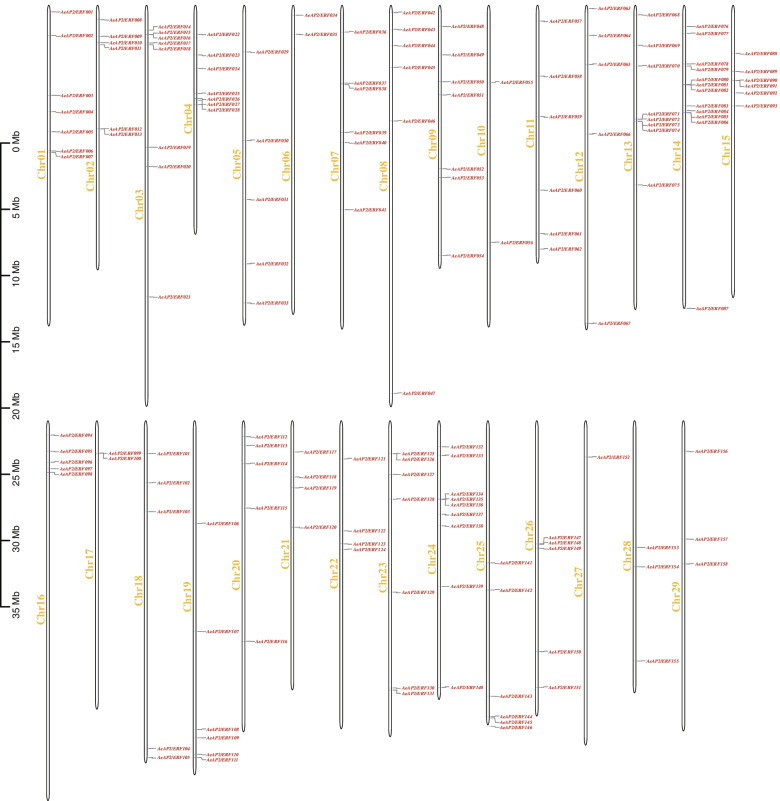
The schematic representations for the distributions of *AP2/ERF* genes in *A. eriantha*

To obtain the intuitionistic distributions of *AP2/ERF* gene family on the chromosomes, a draft was painted. All *AP2/ERF* genes in *A. eriantha* could be detected on the chromosomes and each chromosome had at least one gene (Fig. 4). Most genes were located on chromosomes 14 (twelve genes, 7.5%), followed by chromosomes 24, 13 and 3, consisting of nine, eight and eight genes, respectively. Notably, only one gene (*AeAP2/ERF152*) was found on Chr27. A large number of genes were located on the bottom of chromosome, such as Chr04, Chr08, Chr12, Chr13 and Chr14. However, less genes were near the centromere.

The expansions or contractions of gene family were concerned with gene duplications such as WGD/segmental, tandem, and others including proximal, dispersed and lost. We analyzed the types of duplications in kiwifruit by MCScanX [30]. All types of duplications were found and WGD/segmental account for the largest proportion (76.8%), followed by the type of dispersed (20.6%). Three genes (*AeAP2/ERF073*, *AeAP2/ERF126*, *AeAP2/ERF135*) were tandem duplications. Only one gene (*AeAP2/ERF082*) was proximal. While in *A. chinensis*, more than 90% of the genes were WGD/segmental, only three genes were identified as tandem genes. In addition, nine genes were sought to be singleton (Additional file 3 Table S3). Consequently, WGD/segmental was the crucial factor in the expansion of *AP2/ERF* gene family.

### Collinearity analysis of three putative species and Ka/Ks calculation

**Fig. 5:**
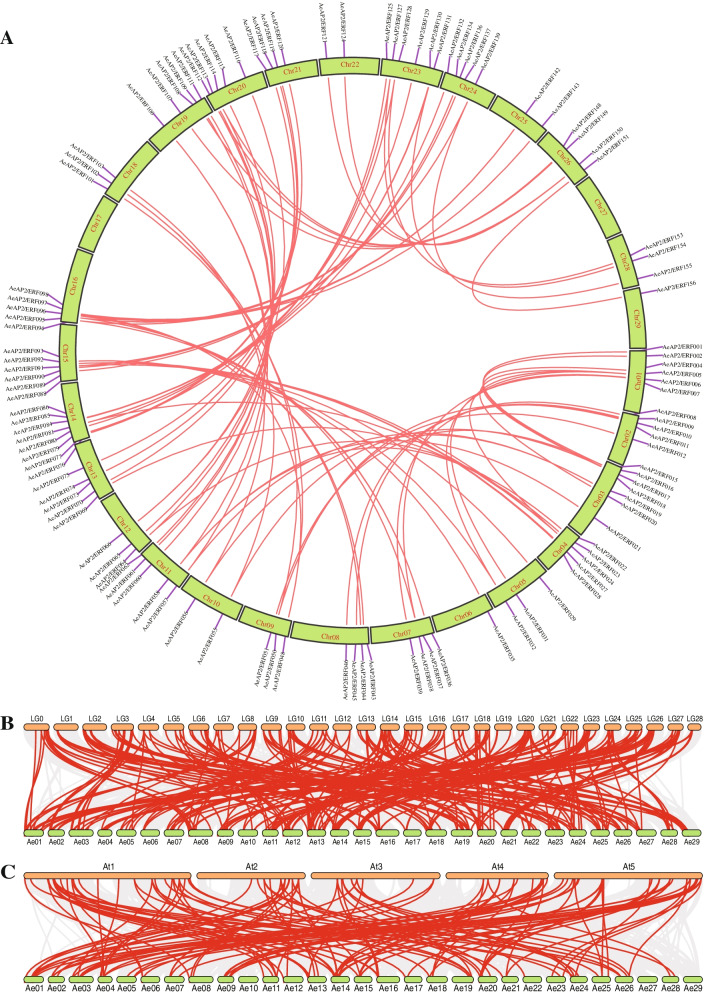
Synteny analysis of *AP2/ERF* gene family in three species. Red line represented the collinear gene pairs in *AP2/ERF* gene family. Chr represents the different chromosome in *A. eriantha*. LG represents the different chromosome in *A. chinensis*. At represents the different chromosome in *A. thaliana*. Synteny analysis of *AP2/ERF* gene family within *A. eriantha* (**A**), between *A. eriantha* and *A. chinensis* (**B**), between *A. eriantha* and *A. thaliana* (**C**)

Intra- and intergenomic relationships demonstrated a favorable collinearity (Fig. 5). A quite number of *AP2/ERF* genes in *A. eriantha* showed a great syntenic relationship with genes from different species. Similarly, compared with analysis in *A. eriantha*, more collinear blocks were clarified and existed between *A. eriantha* and *A. chinensis*, which suggested their kinship was close (Fig. 5B)*.* Specifically, 97 pairs of paralogous genes in *A. eriantha* and 142 pairs in *A. chinensis* were obtained and some genes were involved two or more processes of collinearity evolution. Meantime, 237 gene pairs were confirmed between *A. eriantha* and *A. chinensis* as we expected (Additional file 8 Table S7). Besides, we also detected 75 duplicated gene pairs whose similarities were high than 85%. Among these, about half of gene pairs were collinear (Additional file 9 Fig. S2).

**Fig. 6:**
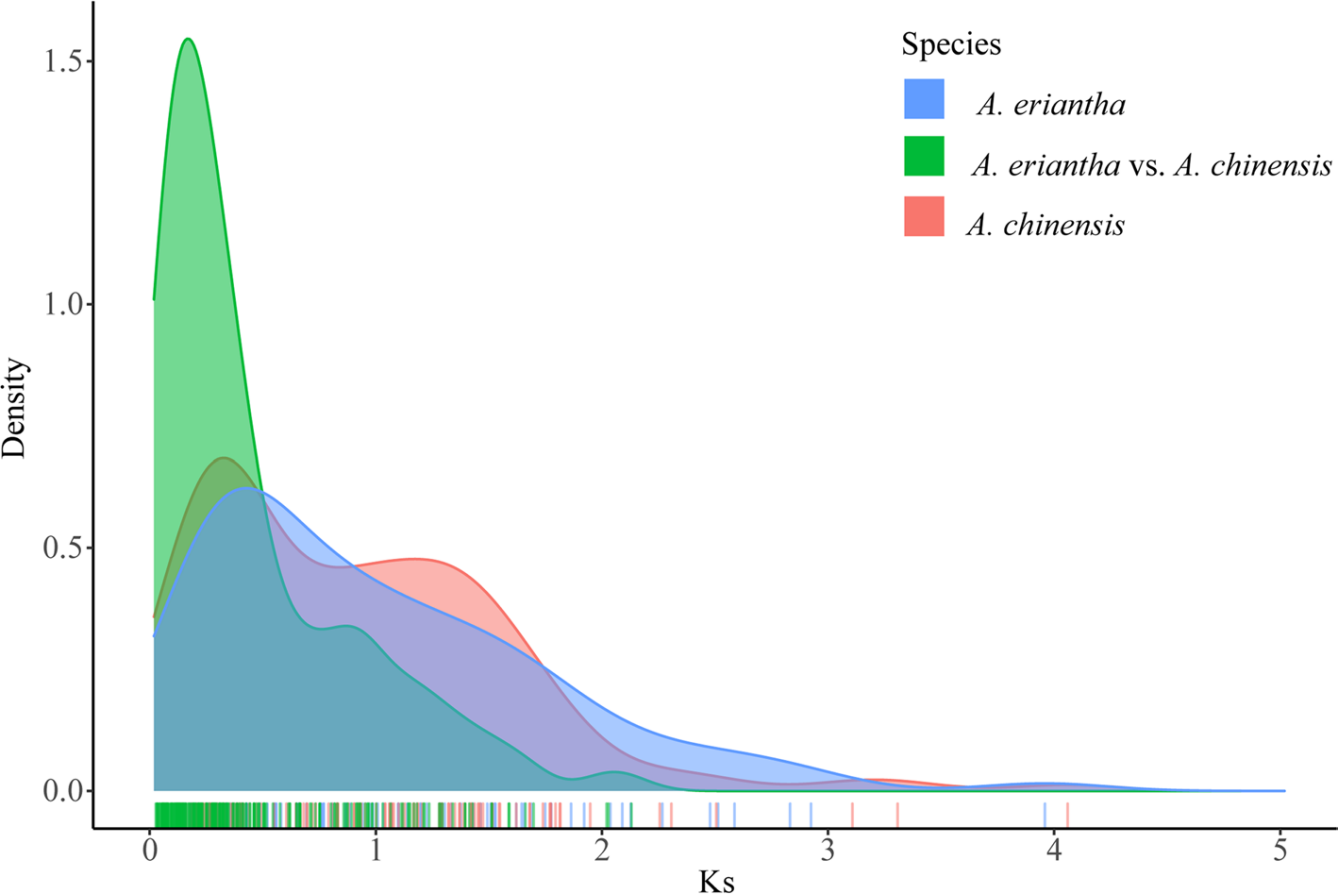
Distribution of the synonymous substitution rate (Ks) between *A. eriantha* and *A. chinensis*

Most Ka/Ks values were less than 1 for *A. eriantha* and *A. chinensis*. However, the ratios of Ka/Ks for three orthologous gene pairs (*AeAP2/ERF029* - *AcAP2/ERF075*, *AeAP2/ERF041* - *AcAP2/ERF106*, *AeAP2/ERF059* - *AcAP2/ERF181*) between *A. eriantha* and *A. chinensis* were more than 1, which suggested that several *AP2/ERF* genes involved in adaptive evolution. Based on homologous gene pairs, we calculated the Ks values and obtained the ranges of peaks of 0.23–0.28 in *A. eriantha*, 0.25–0.30 in *A. chinensis* and 0.01–0.07 between *A. eriantha* and *A. chinensis* corresponding to three divergence time (33–41 MYA, 36–44 MYA and 1.4–10.3 MYA), respectively. Besides, the divergence time of each pair were approximately 4.49–198 MYA, 3.48–134 MYA and 0.26–70 MYA in *A. eriantha*, *A. chinensis* and combination of *A. eriantha* and *A. chinensis*, respectively (Fig. 6; Additional file 8 Table S7).

### The biological functions of *AP2/ERF* genes in kiwifruit flower development

**Fig. 7:**
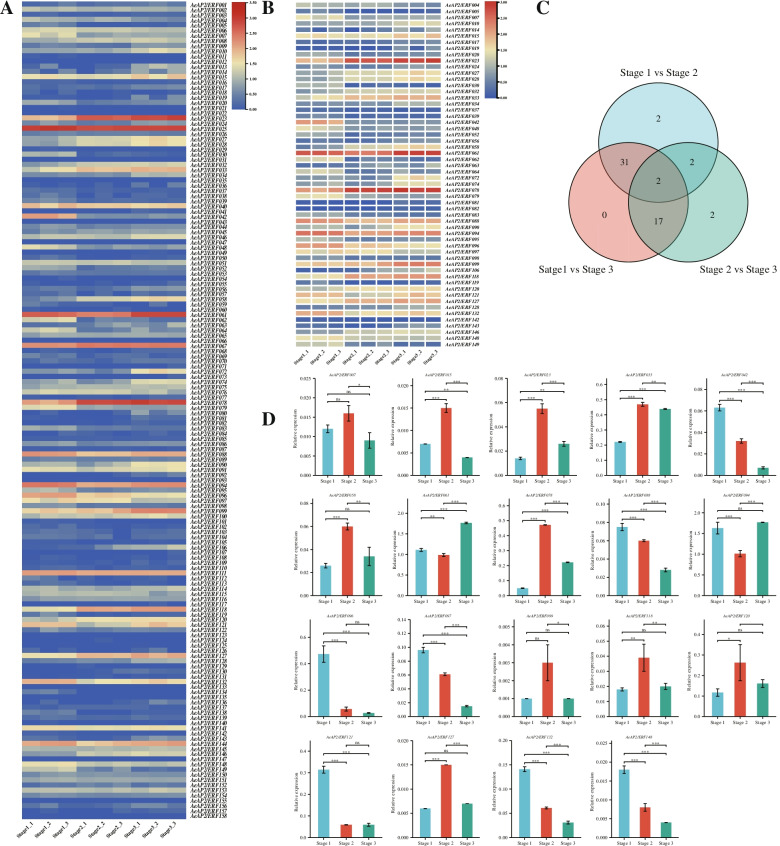
Expression profiles of *AeAP2/ERF*s*.* **A** The expression profile of *AeAP2/ERF* genes in the kiwifruit at different flower developmental stages. **B** The expression profiles of different expression genes in the three contrasts. **C** Venn diagram of DEGs in three combinations. **D** The relative expressions of several *AeAP2/ERF* genes analyzed by qRT-PCR. *: *P* < 0.05; **: *P* < 0.01; ***: *P* < 0.001. The colors blue and red correspond to low and high values of gene expression

To investigate the potential biological functions of *AeAP2/ERF* genes in kiwifruit flower development, flowers in different development stages were prepared for RNA-seq. A total of 232,160,000 clean reads (54 Gb) were obtained with 6 Gb per sample. The mapping rates varied from 84.49 to 85.46% with an average of 84.91% (Additional file 10 Table S8). In addition, we estimated the expression bias of *AeAP2/ERFs* in different flowering periods of *A. eriantha*, and results showed that ten genes (TPM = 0) were not expressed in any stages and three genes (*AeAP2/ERF025*, *AeAP2/ERF061* and *AeAP2/ERF067*) were always abundantly expressed (TPM > 150) (Fig. 7A, B; Additional file 11 Table S9). Interestingly, many of duplicated gene pairs kept the similar expression trends, although their expression levels were not consistent (Additional file 9 Fig. S2). For example, the expression abundances of *AeAP2/ERF023* and *AeAP2/ERF118* gradually increased in the process of flower development. However, there were still a small number of duplicated gene pairs showed the opposite trends as well. For instance, *AeAP2/ERF006* was downregulated, but *AeAP2/ERF033* was upregulated. Besides, 56 DEGs (different expression genes) in *AP2/ERF* family were detected among three combinations (stage1 versus stage 2, stage2 versus stage 3 and stage1 versus stage 3) to explore the primary functions of *AeAP2/ERF*s, and false discovery rate (FDR) < 0.05 and abs (log-fold-change) > 1 served as the standard (Fig. 7C).

To further examine the expression patterns and check the reliability of expression of *AeAP2/ERF*s by RNA-seq, qRT-PCR was performed. Nineteen genes in DEGs with high expressions were randomly selected for validating the accuracy of RNA-seq and primarily confirmed their influence on flower development as well (Fig. 7D). The main expression trends were similar in qRT-PCR and RNA-seq as a whole. These DEGs were expressed significantly in three stages especially between stage 1 and stage 2. Among these DEGs, eleven genes were up-regulate expressed trends as the flowers opening especially from stage 1 to stage 2. On the contrary, expressions of eight genes decreased during the whole process of flower transitions. Thereinto, corresponding with transcriptome data, *AeAP2/ERF061* and *AeAP2/ERF094* showed high expressions in three stage flowers as well. Additionally, in the process of flower development, most of genes exhibited continuous trends (e.g. *AeAP2/ERF042*, *AeAP2/ERF096*), while some genes reached a highest expression level at the stage 2 including *AeAP2/ERF078* and *AeAP2/ERF118*.

## Discussion

AP2/ERF transcription factor family as the essential regulator in many biological processes plays vital roles in the diverse processes including plant growth, fruit ripening and softening, flower development and biotic and abiotic stress responses. Recently, many *AP2/ERF* genes have been confirmed in different species, but the number of *AP2/ERF* genes varies greatly. For instance, 193 *AP2/ERF* genes in *Dactylis glomerata* (~ 1.84 Gb) [31], 134 in tartary buckwheat (*Fagopyum Tataricum*) (~ 489 Mb) [32], 125 in longan (*Dimocarpus longan*) (~ 471 Mb) [33], 200 in *Populus trichocarpa* (~ 394 Mb) [34], and 132 in the grapevine (*Vitis vinifera*) (~ 475 Mb) genome were identified [35]. In this study, we identified 158 *AP2/ERF* genes in *A. eriantha* and 189 in *A. chinensis*. However, 270 *AP2/ERF* genes were identified in *A. chinensis* in the previous study, which might be resulted from different methods, criteria or reference genomes [36]. Although both *A. eriantha* and *A. chinensis* had a similar-sized genome, there is a large difference in gene numbers of *AP2/ERF* family. Compared with *A. eriantha*, more *AP2/ERF* genes were identified in *A. chinensis* (Additional file 3 Tables S3). Thus, the numbers of transcription factors were not tightly associated with the genome sizes, which was mainly caused by the creation of new genes or gene losses in speciation [37]. Plant genome had experienced duplication, polyploidization and transposon insertion in their long evolutionary process. Different evolutionary patterns were likely to produce new genes or new functions [38]. The different number of *AP2/ERF* genes in related species indicates that they probably have extensively expanded in their evolutionary processes. For example, two likely auto-tetraploidization events occurred during the speciation of kiwifruit compared with grape, which might explain why more *AP2/ERF* genes exited in kiwifruit [39]. Collinearity analysis showed that *AP2/ERF* gene family has experienced more duplication events in kiwifruit as well (Fig. 5). Therefore, kiwifruit could be also regarded as a good model to expound the relationship between gene number and genome size in the same genus owing to its complex evolutionary history [40].

Various numbers of introns would result in splice variants and it allowed genes to perform various functions. Besides, it also represents different selective pressures [41]. In the present study, the number of exons in kiwifruit varied greatly and the average number of exons for *A. eriantha* (2.98) was lower than that of *A. chinensis* (3.58) (Additional file 4 Table S4). In other words, our results revealed that there was great difference in the number of introns in whole *AP2/ERF* family in kiwifruit. However, the number of introns within each subfamily varied slightly, and more introns were identified in AP2 subfamily. Compared with AP2 subfamily, genes from ERF and DREB subfamilies had fewer introns, which has been confirmed in *A. thaliana* and Chinese jujube (*Ziziphus jujuba*) [8, 42]. Previous studies revealed that intron was lost slowly during evolution in rice (*Oryza sativa*) and *A. thaliana* [43]. The low rate of intron loss might gradually increase the complexity of evolution patterns, gene functions or the number of genes in gene family in the process of constant change. Consequently, it could be a good explanation for why there are more genes in the ERF and DREB subfamilies where both are less conservative than AP2 subfamily [44].

It has been made clear that some evolutionary events have been increased the members of gene family. While point mutations in exon regions and upstream site of new members could also affect the functions and expression patterns of gene family members [45, 46]. Besides, multifarious motifs and cis-elements appeared in different subfamilies, which also caused differences in functions for various stress responses. For example, in *A. thaliana*, overexpression of TINY, one member of *AP2/ERF* family, increased the survival under the drought stress [47]. However, another gene *AtERF111* was not sensitive to drought but influenced strongly by wounding stress [48]. Even so, as was expected, most closely related family members had similar sections, which indicated they were quite conservative and functional similarity between the AP2/ERF proteins in the same subgroup or in homologous gene pairs. It still needs to be emphasized that even though there were differences, they share some common aspects. For example, they might be sensitive to light signal and likely participated in phytohormone responses induced by abscisic acid (ABA) and jasmonic acid (JA) owing that cis-elements of ABA-responsiveness, MeJA-responsiveness and light-responsiveness.

Notably, the values of Ka/Ks for all gene pairs in *A. eriantha* suggested that they were under the strong negative selection pressures [49]. Additionally, most Ka/Ks values of intron-poor subfamily (ERF and DREB subfamilies) were further away from 1 and lower than the values of AP2 subfamily, suggesting that they experienced stronger selective pressures and needed shorter genes to duplicate easily that took less time [50]. Interestingly, divergence time of *AP2/ERF* genes within *A. eriantha* and between *A. eriantha* and *A. chinensis* were closer to the time of WGD event (Ad-α, 17.7–26.5 MYA) and divergence between *A. eriantha* and *A. chinensis* (~ 11 MYA), suggested that either WGD or species differentiation would have a great influence on the generation, loss and differentiation of genes [26, 27, 39].

Gene functions were deeply associated with their expression patterns [32]. RNA-seq analysis of different stage tissues showed that most genes (104 genes, 65%) were expressed in at least one samples, and one third of genes almost never expressed (Fig. 7A). They might work in a putative condition or have a strong tissue-specific expression. It was also possible that they were pseudogenes. For example, *AeAP2/ERF018* as a representative gene from DREB subfamily was not detected in the present study. Based on the phylogenetic tree, we consider that they had a more recent evolutionary relationship with *AT2G40220.1* (*AtABI4*) in *Arabidopsis*. *AtABI4*, as an important gene related to seed dormancy, was primarily expressed in seeds and rarely detected in other vegetative organs [51]. Our results would provide us a good reference for determining gene functions in kiwifruit. Another homologous gene *AeAP2/ERF011* also followed this rule. Besides, *AtWRI1* were revealed to be involved in seedling development in *A. thaliana* and had a high abundance in seeds [52]. The expression pattern might shed light on the tissue-specific expression of *AeAP2/ERF049* as its homologous gene was also confirmed in tartary buckwheat [32]. *AeAP2/ERF081* was identified as a relative gene with *AT1G63030.1* (*AtDDF2*), but it was distinct in expression profiles. *AtDDF2* most abundantly expressed in rosette leaves and stems, conversely, *AeAP2/ERF081* did not express, which suggested that the functions gradually appeared divergence during evolution. The similar pattern was found in ginger (*Zingiber officinale*) [53]. Additionally, partial genes have high transcription abundance. For instance, the high expression of gene *AeAP2/ERF096* is founded in flowers. According to the previous reports [54], the orthologous gene *AT1G15360.1* (*AtSHIN1*) in *A. thaliana* was just expressed in the flowers and involved in wax biosynthesis. It can be supposed that *AeAP2/ERF096* may perform the same function. Over-expression of the gene might result in glossy leaf phenotype and increase drought tolerance. Interestingly, although both *AeAP2/ERF060* and *AeAP2/ERF096* can be regarded as homologous genes of *AT1G15360.1*, *AeAP2/ERF060* was not detected, suggesting function lose in the evolution process of kiwifruit.

As an important part of plant life history, flowering plays a great role in plant reproduction and survival [55]. Besides, kiwifruit is a perennial vine and breeding early flowering cultivars is an important direction in future breeding. However, few studies were carried out on the molecular mechanism of flowering biology in kiwifruit especially for *AP2/ERF* genes. To intuitively interpret the potential biological functions of *AP2/ERF* gene in flowering, we rechecked several genes identified in RNA-seq using qRT-PCR in *A. eriantha*. About 50% of genes had a positive relation with flowering and mainly reached the peak at stage 2 and seven genes (38%) were negatively correlated. In this study, two genes (*AeAP2/ERF061*, *AeAP2/ERF094*) still showed high expression levels (Fig. 7). Both *AeAP2/ERF061* and *AeAP2/ERF111* were classified into ERF subfamily according to the phylogenetic relationship, their functions were a little different owing that *AeAP2/ERF061* were positive correlated with floral development and *AeAP2/ERF111* negatively controlled flowering although their expressions increased at stage 2. However, their homologue genes *AT1G53910.1* (*AtERF74*) and *AT3G14230.1* played a major role in controlling an RbohD-dependent mechanism or response to various stresses, respectively [56, 57]. Therefore, it is a vital aspect for us to investigate more features of both genes in kiwifruit. Both in *Chrysanthemum morifolium* and *A. thaliana*, *ERF110* was identified to have an influence on flowering [58]. Likewise, the orthologous gene *AeAP2/ERF058* showed increased expression on flowering process. *DDF1* (dwarf and delayed-flowering 1) encoding a member of *AP2/ERF* family was considered to be related to floral development in rice [59]. Its homologues were referred to as *AtDDF1* and *AtDDF2* in *A. thaliana* while they displayed different functions due to duplications [60]. *AeAP2/ERF081* and *AeAP2/ERF102* were found to be closely related to *AtDDF2* and seemed to be not involved in flowering. (Fig. 7B; Additional file 11 Table S9).

Interestingly, *AeAP2/ERF006*, *AeAP2/ERF033* and *AeAP2/ERF051* had a high similarity with *AT5G67180.1* (*AtTOE3*) in *A. thaliana*. *AtTOE3* was associated with floral patterning via interacting with miR172 [61]. However, according to the results of RNA-seq, opposite expressions may indicate various functions in *A. eriantha* owing to function differentiation in their evolution process (decreased expressions of *AeAP2/ERF006* and *AeAP2/ERF051*, and increased expression of *AeAP2/ERF033* in the kiwifruit flower development processes) (Additional file 11 Table S9). Thus, more detailed studies are needed to explore their biological functions in kiwifruit flowering.

## Conclusion

In the present study, we totally identified 158 *AP2/ERF* genes which were divided into four major subfamilies in *A. eriantha.* Synteny analysis showed that these genes demonstrated a favorable collinearity within *A. eriantha,* and many of *AP2/ERF* genes experienced duplication events and were undergoing a purifying selection. Gene structure and protein motif analysis suggested that *AP2/ERF*s in different families were more conservative. Furthermore, our results also showed that one third of *AeAP2/ERFs* were strongly associated with flower transition. Particularly, two genes (*AeAP2/ERF061*, *AeAP2/ERF067*) were expressed abundantly, which indicated that they may act a vital role in plant flower development regulation and flower tissue forming. In summary, the results of this study displayed the first comprehensive analysis of *AP2/ERF* genes in kiwifruit, and it would provide help for screening genes for further functional identification and for genetic improvement of agronomic traits of kiwifruit.

## Methods

### Plant materials

To validate the functions of partial *AP2/ERF* genes in kiwifruit, the flowers in three states (Flower buds, unopened flowers and full-opened flowers) of *A. eriantha* were collected in National Actinidia Germplasm Repository (NAGR) in Wuhan Botanical Garden, the Chinese Academic Science in Hubei province (Fig. 1). To reduce the environmental influences, we collected all samples in 7th May of 2022. One sample was mixed from six individuals of same stage and three repeats were prepared. Then all fresh materials were frozen in liquid nitrogen and stored at − 80 °C for RNA extraction.

### Transcriptome analysis

The flower tissues were sent to BGI company (Wuhan) for transcriptome sequencing. The clean RNA-seq data was mapping onto the reference genome using Hisat2 [62] and generated SAM files were converted to BAM files with SAMtools [63]. Transcriptional abundance and gene expression counts matrix were calculated by featureCounts [64]. The TPM (transcripts per million) value was considered as the expression level and standardized by log10 ^(TPM + 1)^. Furthermore, analysis of differentially expressed genes (DEGs) with FDR < 0.05 and absolute log-fold-change > 1 was performed by edgeR software [65]. The Venn diagram was drawn using jvenn (http://jvenn.toulouse.inra.fr/app/example.html) and the heatmaps were visualized in TBtools [66].

### RNA extraction, and real-time quantitative PCR

All total RNA of samples was exacted by kits (Magen, Hubei, China) according to the manuals. The cDNA was synthesizing using Hifair®II1st Strand cDNA Synthesis Super Mix with the instructions. The cDNA was amplified and quantified by qRT-PCR with 7500FAST (Applied Biosystems) following the manufacturing instructions. The two steps of qRT-PCR were listed as follows: 94 °C for 30s, followed by 45 cycles at 94 °C for 5 s and 60 °C for 30 s. *AeACTIN* were used as internal reference for qRT-PCR analyses based on low variability of expression and stability index values. Expression abundance was calculated using the 2^-ΔΔCt^ method and one-way analysis of variance was used to compare the gene expressions in different periods. The primers sequences were listed in the Additional file 12 Table S10.

### Identification of *AP2/ERF* genes in kiwifruit

We downloaded the genomes of *A. eriantha* and *A. chinensis* in the kiwifruit database (http://kiwifruitgenome.org/) [67]. Subsequently, the update data including 147 AP2/ERF amino acid sequences in *A. thaliana* were retrieved on the Plant Transcription Factor Database (PlantTFDB, http://plntfdb.bio.uni-potsdam.de/v3.0/) [68], and they were used as query sequences to carry out BLASTP program [29] against the whole protein sequences of *A. eriantha* (E-value <1e-10 and identity > = 40). Besides, the Hidden Markov Model profile (PF00847) of AP2/ERF domain in Pfam database (http://pfam.xfam.org/) was obtained for searching the potential genes in kiwifruit by HMMER 3.0 software [28] with default parameters. All sequences confirmed by above two methods were sought to feasible genes. Then, three databases including Pfam (https://pfam.xfam.org/), SMART (http://smart.embl-heidelberg.de/) and NCBI Conserved Domains Database (CDD, https://www.ncbi.nlm.nih.gov/) were used to checking whether these candidate genes comprised of AP2 domain. Sequences contained at least one complete AP2 domain were retained for next analysis. The molecular weight (MW) and isoelectric point (PI) were calculated with the online tool ExPASy (https://web.expasy.org/protparam/). The *AP2/ERF* genes were identified in *A. chinensis* with the same procedures.

### Chromosomal locations and structure analysis of *AP2/ERF* genes

The positions of all genes were extracted from the kiwifruit GFF3 profile, and displayed on the 29 chromosomes via TBtools software [66]. Each exon-intron structure of full coding sequence was presented by iTOL (https://itol.embl.de/).

### Multiple sequence alignments and phylogenetic analysis

Alignments of 497 AP2/ERF protein sequences from *A. thaliana*, *A. eriantha* and *A. chinensis* were conducted by Clustal W [69]. Neighbor-joining (NJ) tree was established using MEGA-X [70] with 1000 replicates of bootstrap. The genes were divided into different clades on the basis of studies on *A. thaliana* [71].

### Conserved motifs and cis-acting regulatory elements analysis

Conserved motifs were identified via Motif-based sequence analysis tools (MEME, http://meme-suite.org/) [72] with the width of motifs changed from 6 to 200. Simultaneously, the selected number of motifs was set to 10.

Cis-acting elements were referred to the motifs used to be testified to deeply influence gene expressions or functions including regulation of plant development, defense of pathogen and adaptation to various environments in the upstream or downstream of the sequences. To preliminarily investigate how to regulate the functions of *AP2/ERF* genes, different types and numbers of cis-elements in *A. eriantha* were predicted via submitting 2,000 bp sequences upstream of the start codon (ATG) in genomic DNA sequences to Plant CARE database (http://bioinformatics.psb.ugent.be/webtools/plantcare/html/) [73]. All consequences were visualized with TBtools [66].

### Analysis of gene duplication events and Ka/Ks calculation

MCScanx software [30] was used to detect the collinearity and duplication events among the genomes of *A. thaliana* and two related species of *Actinidia.* Likewise, synteny between *A. eriantha and A. chinensis* genomes was analyzed.

Ks (synonymous substitution rate), Ka (nonsynonymous substitution rate) and Ka/Ks were calculated to understand whether the *AP2/ERF* family was undergoing the selections during the evolution process. Briefly, 1 stands for neutral selection; less than 1 represents for negative selection and more than 1 means positive selection [49]. The ratios of synonymous (Ks) and non-synonymous (Ka) nucleotide substitutions (Ka/Ks) between homologous gene pairs were also calculated via Simple Ka/Ks Calculator module in TBtools [66]. Additionally, the divergence time was calculated using the formula T = Ks/2r. Thereinto, Ks referred to the ratio of synonymous and r was equal to 3.39 × 10^− 9^ synonymous substitutions per site per year [26]. The syntenic analysis maps were also drawn using the Dual Systeny Plotter software in TBtools [66]. Distribution of the synonymous substitution rate (Ks) was shown by ggplot2 packages [74].

## Supplementary Information

**Additional file 1: Table S1.** The protein sequences and coding sequences of the *AP2/ERF* gene family in *A. eriantha* and *A. chinensis*.

**Additional file 2: Table S2.** The detailed information of exited AP2 domain in listed *AP2/ERF* genes in this study.

**Additional file 3: Table S3.** The basic information of *AP2/ERF* gene family in *A. eriantha* and *A. chinensis*.

**Additional file 4: Table S4.** The gene structures of *AP2/ERF* gene family in *A. eriantha* and *A. chinensis*.

**Additional file 5: Table S5.** The amino acid sequence of motif and annotation in AeAP2/ERF proteins

**Additional file 6: Fig. S1.** Distributions of all cis-elements in *AP2/ERF* genes in *A. eriantha.*

**Additional file 7: Table S6.** Cis-elements in the promoters of putative *AP2/ERF* genes in *A. eriantha*.

**Additional file 8: Table S7.** The results of Ka/Ks calculations of *AP2/ERF* homologous gene pairs between *A. eriantha* and *A. chinensis*.

**Additional file 9: Fig. S2.** The expression heatmap of duplicated gene pairs in *A. eriantha*. Each line represents one duplicated gene pair.

**Additional file 10: Table S8.** Summary of the raw sequencing data and mapping rate of *A. eriantha*.

**Additional file 11: Table S9.** The TPM values of different flower developments in RNA-seq of *A. eriantha*.

**Additional file 12: Table S10.** Primer sequences of *AP2/ERF* genes in *A. eriantha* for qRT-PCR.

## Data Availability

The clean RNA-seq reads were deposited at NCBI SRA database (http://www.ncbi.nlm.nih.gov/sra) under the Bioproject accession number PRJNA855574.

## Abbreviations

AP2/ERF: APETALA 2/Ethylene Responsive Element Binding Factor
DREB: Dehydration-responsive element-binding protein
HMM: Hidden Markov Model
DEGs: Different expression genes
qRT-PCR: Real-time quantitative PCR
MCScanx: Multiple Collinearity Scan toolkit X
