# Supplementary material for: Genome-wide identification and characterization of AP2/ERF gene superfamily during flower development in Actinidia eriantha
Source: BMC Genomics. 2022 Sep 13;23:650. doi: 10.1186/s12864-022-08871-4 (PMC9469511; doi:10.1186/s12864-022-08871-4)
Supplement: Supplementary file 5 — Additional file 5: Table S5. The amino acid sequence of motif and annotation in AeAP2/ERF proteins [file 12864_2022_8871_MOESM5_ESM.docx]

Table S5 The amino acid sequence of motif and annotation in AeAP2/ERF proteins

| Motif | sites | Amino acid sequence of motif | Width  (aa) | Specific  domain/region |
| --- | --- | --- | --- | --- |
| Motif 1 | 154 | GTFDTAEEAARAYDEAAIKLRGPKAVLNF | 29 | AP2 domain |
| Motif 2 | 158 | VRQRPWGKWVAEIR | 14 | AP2 domain |
| Motif 3 | 123 | EPRKKARVWL | 10 | - |
| Motif 4 | 22 | QGGYDTEEAAARAYDLAAJKYRGVDADINFPLSDY | 35 | AP2 domain |
| Motif 5 | 14 | EEDLEZMKNLTKEEYVAILRRKSSGFSRGSSKYRGV | 36 | - |
| Motif 6 | 24 | PDSAHSLPVPASTSPRDIQAAAAKAAAAF | 24 | - |
| Motif 7 | 120 | DGPGSRHSKYRG | 12 | - |
| Motif 8 | 13 | AGGSEFVDEEAJFDMPNLLVNMAEGMLLSPPRL | 33 | - |
| Motif 9 | 5 | AKLRKCCKSPSPSLTCLRLDPENSRIGVWQKRAGPRSDSKWVMTVQLGKK | 50 | - |
| Motif 10 | 7 | HRWTGRYEAHLWDNSCRREGQSRKGR | 26 | - |
