# Supplementary figures and images for: Genome-wide identification and characterization of AP2/ERF gene superfamily during flower development in Actinidia eriantha
Source: BMC Genomics. 2022 Sep 13;23:650. doi: 10.1186/s12864-022-08871-4 (PMC9469511; doi:10.1186/s12864-022-08871-4)

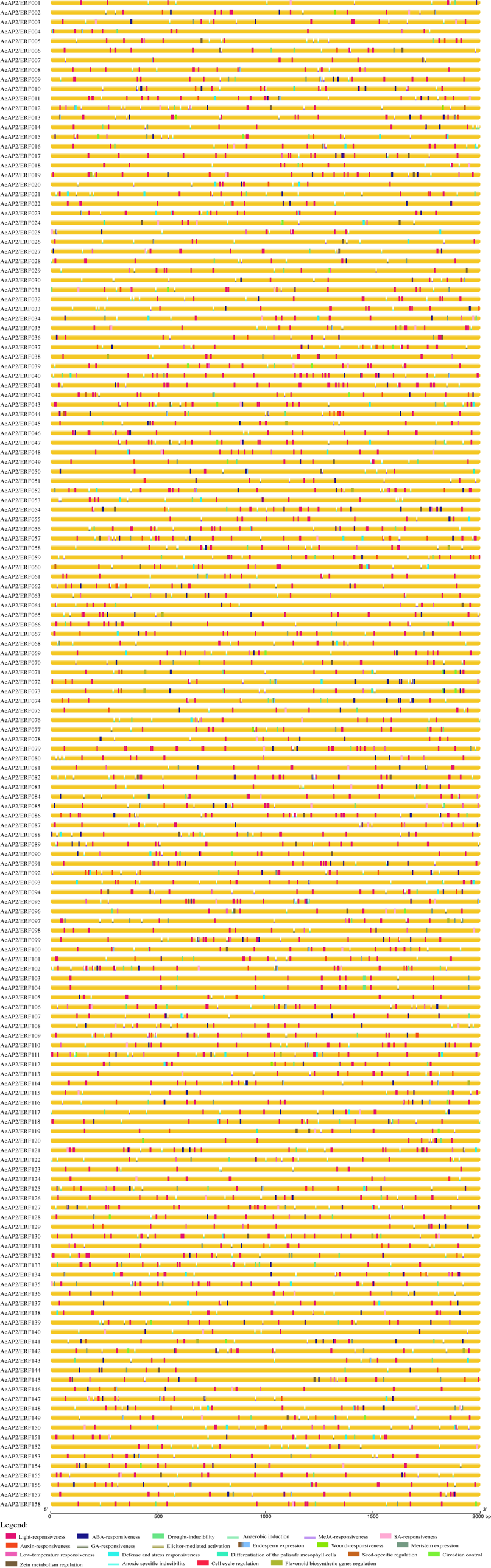

Supplement: Supplementary file 6 — Additional file 6: Fig. S1. Distributions of all cis-elements in AP2/ERF genes in A. eriantha. [file 12864_2022_8871_MOESM6_ESM.jpg]

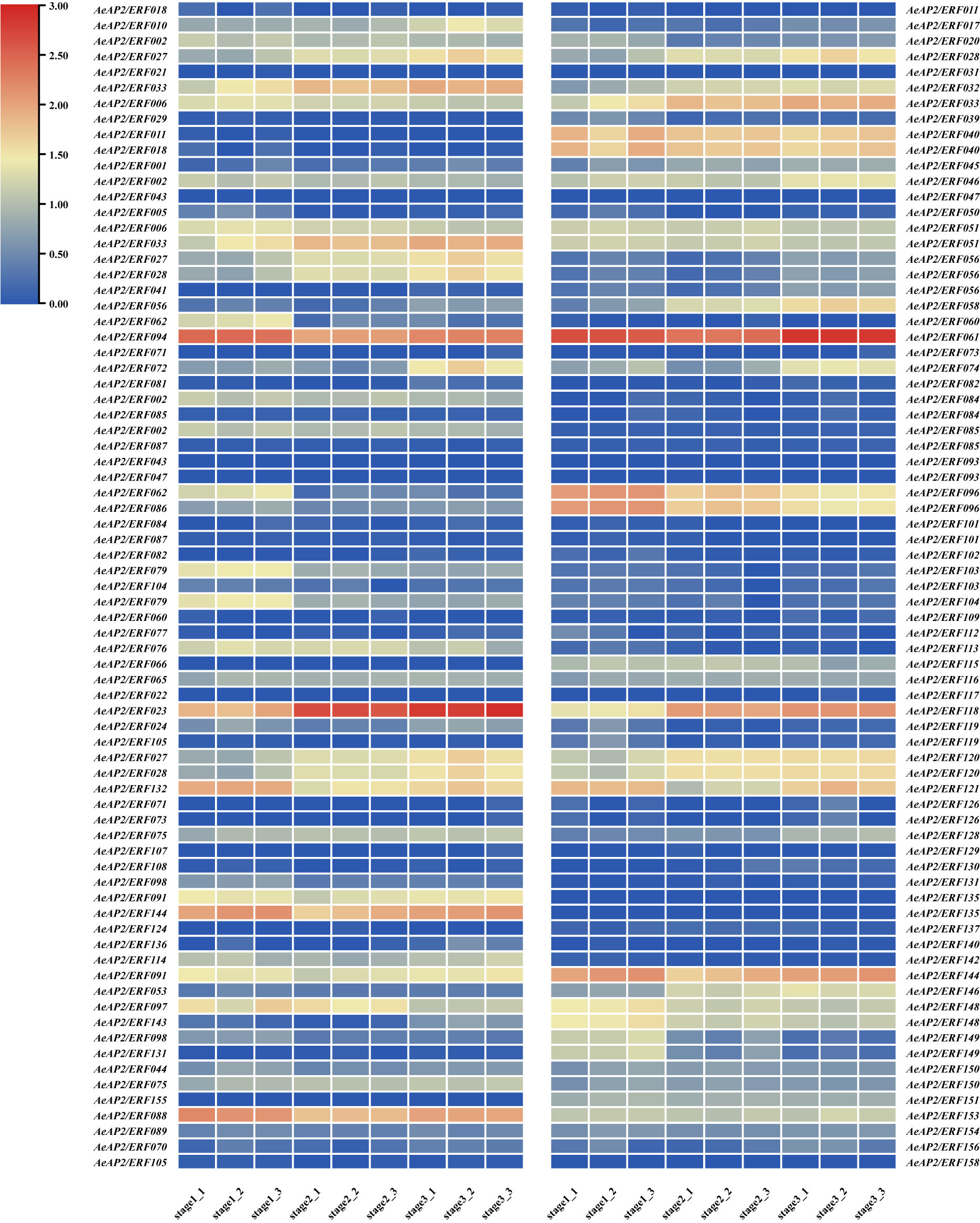

Supplement: Supplementary file 9 — Additional file 9: Fig. S2. The expression heatmap of duplicated gene pairs in A. eriantha. Each line represents one duplicated gene pair. [file 12864_2022_8871_MOESM9_ESM.tif]
